# Phenotypes and Molecular Mechanisms Underlying the Root Response to Phosphate Deprivation in Plants

**DOI:** 10.3390/ijms24065107

**Published:** 2023-03-07

**Authors:** Meiyan Ren, Yong Li, Jianshu Zhu, Keju Zhao, Zhongchang Wu, Chuanzao Mao

**Affiliations:** 1 State Key Laboratory of Plant Physiology and Biochemistry, College of Life Sciences, Zhejiang University, Hangzhou 310058, China; 2 Hainan Institute, Zhejiang University, Yazhou Bay Science and Technology City, Sanya 572100, China

**Keywords:** *Arabidopsis thaliana*, *Oryza sativa*, root architecture, phosphorus, phosphate starvation response

## Abstract

Phosphorus (P) is an essential macronutrient for plant growth. The roots are the main organ for nutrient and water absorption in plants, and they adapt to low-P soils by altering their architecture for enhancing absorption of inorganic phosphate (Pi). This review summarizes the physiological and molecular mechanisms underlying the developmental responses of roots to Pi starvation, including the primary root, lateral root, root hair, and root growth angle, in the dicot model plant *Arabidopsis thaliana* and the monocot model plant rice (*Oryza sativa*). The importance of different root traits and genes for breeding P-efficient roots in rice varieties for Pi-deficient soils are also discussed, which we hope will benefit the genetic improvement of Pi uptake, Pi-use efficiency, and crop yields.

## 1. Introduction

Phosphorus (P), an essential macronutrient for plant growth, plays a central role in a variety of vital processes, including energy generation, photosynthesis, glycolysis, respiration, and nucleic acid and membrane lipid biosynthesis [1,2]. Inorganic phosphate (Pi) is the predominant form of P that can be absorbed directly by plants [3]. Due to its low mobility and high fixation rate, the availability of Pi in soils is very low, although the total P content in most soils is high. The low-Pi availability in soils and the imminent scarcity of the non-renewable Pi rock make Pi deficiency a major limitation on crop production [4]. Improving crop Pi uptake and Pi-use efficiency will be an important component of any comprehensive strategy to achieve sustainable P use. Roots, the primary organ by which plants sense and explore Pi, have a dominant influence on Pi uptake and plant yield [5,6]. Many papers have reviewed the changes in root architecture in response to Pi limitation in the dicotyledonous model plant *Arabidopsis thaliana*; however, few have reviewed the root architecture response to Pi limitation in monocotyledonous plants. Rice (*Oryza sativa*) is one of the most important food crops in the world; developing varieties with Pi-efficient root system under low P is a valid approach to enhance low Pi tolerance and yield in rice; thus, understanding the mechanisms of root remodeling under Pi deficiency in this species is of great importance. Increasing studies indicate that Arabidopsis and rice have different root architectures and regulatory mechanisms in response to Pi deprivation; however, the similarities and differences in the phenotype and molecular mechanism of root response to Pi deficiency between Arabidopsis and rice are still unclear. This review compares the conserved and divergent aspects of the root response to Pi deprivation in Arabidopsis and rice with a focus on the integrated physiological and molecular mechanisms, which we hope will benefit the genetic improvement of crop Pi efficiency.

## 2. Root Response to Pi Deficiency in Arabidopsis and Rice

### 2.1. Root Morphology of Arabidopsis and Rice

Arabidopsis has a taproot system, which consists of a single thick primary root (PR) and multiple lateral roots (LRs), all of which have surface area-enhancing root hairs (RHs) [7]. The PR originates from the embryo, while LRs emerge from the PR and other LRs through LR primordia that initiate from pericycle founder cells [8]. RHs are differentiated, initiated, and elongated from RH-destined epidermal H-cells located on the PR and LRs and play roles in absorbing nutrients and water, as well as anchoring the root into the soil [8,9]. LRs tend to grow from the PR at a specific angle, named the root growth angle (RGA), allowing the roots to explore the soil nutrients and water more effectively [10]. The RGA is an important component of the Arabidopsis root system, regulating and optimizing its radial spread.

In contrast to Arabidopsis, rice bears a fibrous root system consisting of a PR and multiple crown roots (CRs, or adventitious roots), LRs, and RHs. CRs are shoot-borne roots that initiate from parenchyma cells at the base of the stem [11]. LRs in rice are generated on the PR and the CRs. RHs emerge from the PR, CRs, and LRs, accounting for a large portion of the root surface area [12]. Unlike in Arabidopsis, the rice PR is only important at the early stage of seedling development, after which its anchoring role is gradually taken on by the CRs. The RGA is an important component of the root system in rice, just as it is in Arabidopsis, functioning to ensure roots are properly distributed in the soil to explore the soil nutrient and water resources [13]. The RGA in rice mainly depends on the angle between the CRs and the direction of gravity, although the angle between the LRs and the CRs also contributes to the overall RGA. In Arabidopsis, the RGA mainly depends on the angle between the LRs and the direction of gravity (or the PR).

### 2.2. Root Response to Pi Deprivation in Arabidopsis and Rice

In most studies in Arabidopsis, Pi deficiency inhibits PR growth and enhances LR and RH growth to increase the total root surface area [14]. Under Pi deprivation, the reduction of cell elongation in the PR elongation zone is followed by defects in the stem cells and the progressive exhaustion of meristematic cells in its meristematic region, resulting in the growth inhibition of the PR [15]. Further studies indicated that iron (Fe) plays an important role in the Pi-dependent inhibition of PR growth. The degree of PR inhibition in Arabidopsis was positively correlated with the levels of Fe, and this Fe-dependent inhibition could be abolished in a Pi-limited medium by adding Fe scavengers [16]. Later, Müller et al. reported that the Fe^3+^-dependent accumulation of callose in the apoplast of the stem cell niche contributed to the loss of stem cells and the resulting meristematic cell exhaustion [17]. Mora-Macías et al. further indicated that malate-dependent Fe accumulation in the apoplast triggers the Pi-dependent differentiation of meristematic cells in the root apical meristem [18]. In addition, Zheng et al. reported that blue light is not only required but also sufficient to inhibit PR growth through a blue-light-mediated photo-Fenton reaction that converts Fe^3+^ to Fe^2+^ under Pi deficiency [19]. Interestingly, a recent study found that the degree of PR inhibition induced by Pi deficiency is not linked to the level of Fe accumulated in the root apical meristem or the elongation zone [20]. Taken together, these findings show that Fe accumulation is a critical checkpoint in the inhibition of PR growth under Pi deprivation.

By contrast, hormones play irreplaceable roles in altering LR and RH growth and the RGA in response to Pi starvation [21,22]; for example, the Pi deficiency–induced LR growth in wild-type Arabidopsis was not observed in strigolactone (SL)-deficient mutants [23]. Low Pi resulted in larger RGAs for the LRs in Arabidopsis in an auxin-dependent manner [24]. Auxin also promotes RH growth in Pi-limited Arabidopsis roots [25]. In addition, sucrose also participates in the root development response to Pi starvation [26,27]. Due to limited space, we will not discuss it here as it has been well documented in the reviews [26,27].

Compared with Arabidopsis, the Pi starvation response in rice is quite diverse and complicated. In hydroponic systems, rice plants showed either an enhanced or unaffected PR growth under Pi-deficient conditions, depending on the genetic background [28]; however, some studies contrastingly reported that slight Pi deficiency promotes root growth, while mild and severe Pi deficiency suppresses root growth in this crop [14]. Similarly, an increase or no change in LR elongation is reported in hydroponic culture among different rice varieties grown under low P [28,29]. Using a sand culture system, which mimics realistic soil Pi availability, Vejchasarn et al. showed that low Pi reduced LR density and length but increased RH length and density in all 15 tested rice varieties [30]. Interestingly, Nestler et al. reported that rice plants grown in low-Pi soil produced shorter RHs than those grown in Pi-sufficient soil [31]. In addition, a recent study indicated that Pi deficiency induced a shallower root system in rice by modifying root gravitropism in the soil [32].

To date, the root morphology and architectural response to Pi deficiency are relatively clear in Arabidopsis under controlled growth conditions, while it is still ambiguous in rice. The complex interactions between the growth conditions and genetic variability make it difficult to study the root architectural response under low-Pi soil conditions. With the development of modern technology, the precise study of root morphology and the architectural response to Pi deficiency in the soil should quickly progress.

## 3. Molecular Mechanisms Underlying the Root Response to Pi Deficiency

### 3.1. Molecular Mechanisms Underlying PR (and CR in Rice) Growth under Pi Deprivation in Arabidopsis and Rice

#### 3.1.1. Fe Accumulation Is Responsible for Inhibiting PR Growth under Pi Deficiency

The molecular mechanisms underlying the Pi deprivation-mediated inhibition of PR growth in Arabidopsis are relatively clear. Fe accumulation is known to impact PR growth; for example, malate-dependent Fe accumulation contributes to the inhibition of PR growth under Pi deficiency in Arabidopsis [33]. Based on forward genetics, a series of genes involved in this process were identified through screening mutants with longer or shorter roots than the wild type under Pi-deficient conditions. The *low phosphate root 1* (*lpr1*) mutant had a wild-type phenotype under Pi-replete conditions but displayed less inhibited PR growth and reduced Fe^3+^ accumulation in the root apoplast under Pi-limited conditions [34]. Further study indicated that AtLPR1 belongs to a multicopper oxidase family, possessing ferroxidase activities that convert Fe^2+^ to Fe^3+^ in the apoplast [17,34]. AtLPR2, a close paralog of AtLPR1 in Arabidopsis, also functions as a ferroxidase but plays a less important role in Pi deficiency–induced PR inhibition than AtLPR1 [35]. The *phosphate deficiency response 2* (*pdr2*) mutant over-accumulated Fe^3+^ in the root apoplast, causing an enhanced inhibition of PR growth under low-Pi conditions [36]. *AtPDR2* encodes a P5-type ATPase, which is thought to control AtLPR1 biogenesis or AtLPR1-reactant Fe availability in the apoplast [36].

Later, ALUMINUM-ACTIVATED MALATE TRANSPORTER 1 (AtALMT1) was reported to regulate the rapid inhibition of cell elongation in the transition zone in response to low-Pi availability by exuding malate [33,37]. The zinc-finger transcription factor SENSITIVE TO PROTON RHIZOTOXICITY 1 (AtSTOP1) acts as the master regulator for the Pi deficiency-mediated inhibition of PR growth by accumulating in the nucleus and directly upregulating the expression of *AtALMT1* under low-Pi conditions [33,38]. *ALUMINUM SENSIRIVE 3* (*AtALS3*), encoding the transmembrane domain of a putative ABC (ABC-binding cassette) transporter, and *SENSITIVE TO AL RHIZOTOXICITY 1* (*AtSTAR1*), encoding the nucleotide-binding domain of the same ABC transporter, have also been reported to be involved in PR inhibition under Pi deficiency [39]. The *atals3* and *atstar1* mutants showed an enhanced accumulation of Fe in the root apoplast, which resulted in their hypersensitive PR inhibition phenotypes under Pi deficiency [39]. AtALS3 and AtSTAR1 form a typical ABC transporter protein complex. Under Pi-deficient conditions, the activity of this AtALS3/AtSTAR1 transporter was reduced, promoting the accumulation of AtSTOP1 in the nucleus and, thereby, increasing the expression of *AtALMT1* to inhibit the growth of the PR [20]. A recent study found that low Pi triggers the uptake of ammonium, causing the rapid acidification of the root surface. This rhizosphere acidification triggers the accumulation of AtSTOP1 in the nucleus, which then triggers the AtALMT1-mediated excretion of malate to start the root developmental responses [40]. In addition, Fe^2+/3+^ were reported to directly increase the accumulation of AtSTOP1 in the nucleus by inhibiting its proteasomal degradation in a pH-dependent manner [41]. A possible mechanism by which low Pi inhibits PR growth in Arabidopsis, proposed by Dong Liu [14], is that P deficiency results in a low pH and the accumulation of Fe, which stimulates AtSTOP1 accumulation in the nucleus and upregulates the expression of *AtALMT1.* This, in turn, promotes the excretion of malate into the root apoplast, where the malate–Fe^3+^ complex is formed when AtLPR1 is functional. The low-pH environment and blue light together promote the conversion of malate–Fe^3+^ into Fe^2+^ via a photo-Fenton reaction, after which Fe^2+^ reacts with H_2_O_2_ to produce hydroxyl radicals and Fe^3+^ via a Fenton reaction. This Fe redox cycle causes the continual production of hydroxyl radicals, which inhibit PR growth [14].

Recently, more genes have been found to be involved in this AtSTOP1–AtALMT1 signaling network; for example, *AtSIZ1* (*SAP and Miz 1*), encoding a SUMO E3 ligase, negatively regulates AtSTOP1 signaling and the expression of *AtALMT1* [42,43]. In addition, the transcription factor AtWRKY33 has been reported to function as a negative regulator for Pi deficiency-inhibited PR growth by regulating the expression of *AtALMT1* and subsequent Fe^3+^ accumulation in Arabidopsis root tips [44]. AtWRKY33-deficient mutants were more sensitive to low Pi, producing a shorter PR than the wild type under Pi-limited conditions.

Unlike Arabidopsis, rice plants exhibit more PR and CR growth when grown in Pi-deficient conditions, the extent of which depends on their genetic background. This contrast suggests that rice and Arabidopsis use different mechanisms to control the root response to Pi limitation. Low Pi increased the expression of the five *AtLPR1* homologous genes in rice [45,46], including *OsLPR5*, which was previously functionally characterized as a ferroxidase encoding gene [47]. The overexpression of *OsLRP5* increased the Fe^3+^ concentration in the xylem sap, and the total Fe content in the rice roots and shoots. *OsPDR2*, a homolog of Arabidopsis *AtPDR2*, plays an important role in root development and Pi homeostasis in rice [48]; however, the mutation of *OsLRP5* and *OsPDR2* resulted in shorter PRs and CRs than the wild type, irrespective of the Pi conditions [47,48]. In addition, *OsWRKY74*-overexpressing plants produced a longer PR and accumulated more Fe than the wild-type plants under Pi-limited conditions, whereas the *OsWRKY74* RNAi plants produced a shorter PR [49]. This result indicates that *OsWRKY74* is involved in the Pi and Fe starvation responses and may act as an integrator of these stress response pathways in rice. It also highlights the important roles of WRKY family members in Pi-dependent PR growth in Arabidopsis and rice.

#### 3.1.2. Plant Hormone Signaling Pathways Are Involved in PR (and CR in Rice) Growth under Pi Deficiency

Auxin plays fundamental roles in regulating Pi-dependent PR (and CR in rice) growth. The *osaux4* (*rice homolog of Arabidopsis auxin resistant 4*) mutant, which has an aberrant auxin content and distribution, is insensitive to Pi starvation, suggesting that OsAUX4 positively regulates PR and CR elongation in response to low Pi [50]. The auxin efflux transporter PIN-FORMED 1b (OsPIN1b) affects PR and CR elongation by regulating the root apical meristem activity under low-Pi conditions [51]. In addition, the loss-of-function mutants of *AUXIN RESPONSE FACTOR 12* (*OsARF12*) and *OsARF25* showed a reduced sensitivity to Pi deficiency in the PR and CR response [52]. These results suggest that auxin mediates PR and CR remodeling in response to Pi deficiency in rice. The attenuated sensitivity of Pi deficiency–inhibited PR growth in mutants of the auxin influx transporter gene *AtAUX1* or in triple mutants of the auxin receptor gene *TRANSPORT INHIBITOR RESPONSE 1* (*AtTIR1*) and its closest paralogs, encoding the auxin signaling F-box proteins 2 and 3, highlights the important role of auxin in this process in Arabidopsis [21,53].

In Arabidopsis, AtSIZ1 regulates PR remodeling under Pi deficiency in an auxin-dependent way [54,55]. The expression of the auxin-responsive genes was unchanged in the *atsiz1* mutant, but under Pi-starved conditions it accumulated auxin earlier in its roots than did the wild-type plants. By contrast, this auxin-dependent root architecture alteration under Pi deficiency was not observed in the *ossiz1* and *ossiz2* mutants in rice, the homologs of Arabidopsis *SIZ1* [56,57]. The *ossiz1* mutant had shorter PRs and CRs than the wild type, independent of the Pi status in the environment. Ding et al. investigated the role of auxin in Pi-dependent root elongation in rice using three varieties [58], revealing that Pi-starved Tongjing981 (TJ981) and Zhendao99 (ZD99) had a longer PR than they did under normal Pi, whereas Zhenghan6 (ZH6) produced a shorter PR under Pi starvation than it did under normal Pi. They further indicated that Pi deficiency increased the indole-3-acetic acid (IAA) contents in TJ981 and ZD99 but decreased the IAA content in ZH6. This increased IAA content induced the expression of expansin genes and the activation of expansin proteins, leading to cell wall relaxation and root elongation in TJ981 and ZD99. By contrast, the expansin genes were downregulated in Pi-starved ZH6, resulting in its shorter PR. Auxin is therefore involved in regulating Pi-dependent PR growth in both Arabidopsis and rice.

Ethylene is another important plant hormone regulating PR growth in response to Pi starvation [59]. *HYPERSENSITIVE TO PI STARVATION 4* (*AtHPS4*), a new allele of *SABRE* encoding an important regulator of cell expansion, is known to respond to Pi starvation by interacting with ethylene signaling [59,60]. The *athps4* mutant displayed a shorter PR than the wild type under high-Pi conditions, but also exhibited a stronger inhibition of PR growth under low-Pi conditions. Silver ions (Ag^+^), an inhibitor of ethylene action, suppressed this response, confirming the important role of ethylene in HPS4-dependent PR inhibition in response to low Pi. AtHPS7 (a tyrosylprotein sulfotransferase), AtHPS3, and ETHYLENE OVERPRODUCTION 1 (AtETO1) also regulate Pi-dependent PR growth through the ethylene signaling pathway [61,62,63]. The *athps7* mutant displayed an exaggerated inhibition of PR growth, accompanied by an elevated expression of ethylene biosynthesis genes, compared with the wild-type plants under low-Pi conditions. Similarly, low Pi inhibited the PR growth of the *athps3* mutant, which was attributed to the overproduction of ethylene under the regulation of AMINOCYCLOPROPANE-1-CARBOXYLATE SYNTHASE 4 (AtACS5), the rate-limiting enzyme in ethylene biosynthesis. Recently, the transcription factor Arabidopsis NAM/ATAF/CUC protein 44 (ANAC044) was illustrated to alter the PR length in Arabidopsis under Pi starvation conditions by increasing the cell wall Pi reutilization in an ethylene-dependent manner [64]. The *anac044* mutant produced a longer PR than the wild type under Pi-limited conditions by regulating the expression of ethylene production genes. The role of ethylene in regulating PR growth in response to Pi starvation in rice has not been reported yet.

Gibberellin (GA) also participates in the Pi deficiency-mediated inhibition of PR growth [65]. GA stimulates growth by promoting the destruction of DELLA proteins in the 26S proteasome. The DELLA-deficient plants do not exhibit the Pi starvation–induced PR growth inhibition observed in the wild type [66]. AtMYB62, an R2R3-type transcription factor that regulates the GA metabolism and signaling in Arabidopsis, affects the PR growth response to Pi availability [65]. *AtMYB62*-overexpressing plants have shorter PRs than wild-type plants under Pi-replete conditions but showed comparable PR lengths under Pi-limited conditions. In rice, OsMYB1 affects both Pi starvation signaling and GA biosynthesis, with the *osmyb1* mutant producing a longer PR than the wild type under Pi starvation, independent of GA [67].

Small signaling peptides are also involved in the Pi-dependent inhibition of PR growth. The small signaling peptide CLAVATA3/EMBRYO SURROUNDING REGION 14 (AtCLE14) has been proven to play a major role in the exhaustion of the meristematic cells under Pi-limited conditions [68]. The expression of *AtCLE14* in the proximal meristem region was enhanced via Fe mobilization in the root apical meristem under the control of AtLPR1/AtLPR2. Low-Pi conditions induced *AtCLE14* expression, with the resulting peptide perceived by the receptors CLAVATA2 (AtCLV2) and PEP1 RECEPTOR 2 (AtPEPR2), leading to the downregulation of SHORT ROOT/SCARECROW (AtSHR/AtSCR), WUSCHEL-RELATED HOMEOBOX5 (AtWOX5), and the PIN proteins, which are involved in the maintenance of the root stem cell niche. This resulted in root meristem exhaustion and the inhibition of PR growth.

In addition to the above regulatory mechanisms, CASEIN KINASE 2 (AtCK2) was recently suggested to trigger Pi starvation-induced stem cell exhaustion and thus inhibit PR growth by activating the DNA damage–response pathway in Arabidopsis [69]. Whether OsCK2 and its interactors affect Pi-dependent root exhaustion in rice has yet to be investigated, however. On the other hand, the *leaf tip necrosis1* (*ltn1*) mutant of the putative rice orthologous gene of *PHOSPHATE 2* (*PHO2*) in Arabidopsis had longer PR and CRs than the wild type under Pi-limited conditions; however, there was no difference in PR and CR length between *ltn1* and the wild type under Pi-replete conditions [70]. The *ltn1* mutant had a greater Pi content in the shoot but lower Pi content in the root compared with the wild type, suggesting that this decreased Pi content in the roots of *ltn1* may contribute to its longer PR and CRs under Pi deficiency. However, it has not been determined yet whether the root of Arabidopsis *pho2* responds to Pi deficiency.

**Figure 1:**
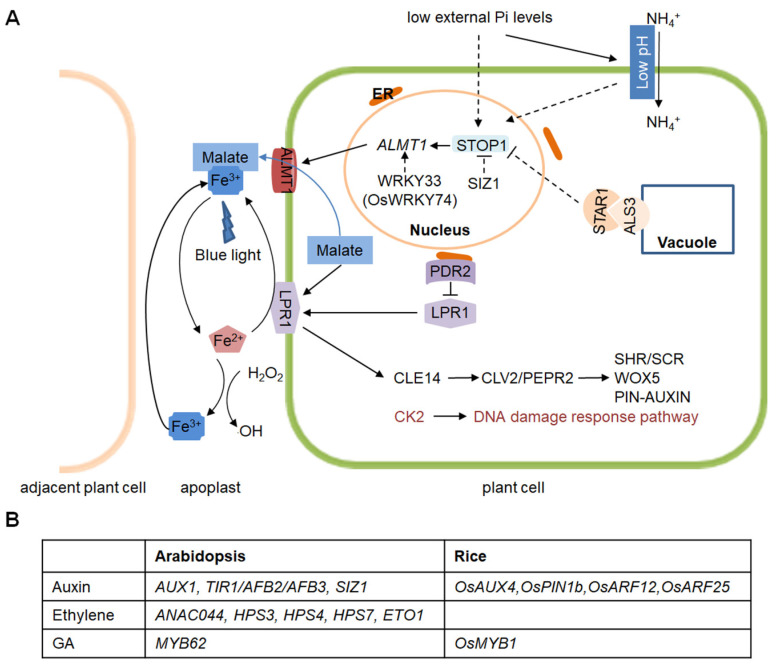
A working model of the molecular mechanisms by which Pi starvation inhibits PR growth in Arabidopsis and rice. (**A**) The working model for Pi-dependent PR growth (rice homologous genes are labeled). The model is modified from that of Liu et al. (2021), with updated genes in Arabidopsis and rice. Arrows with solid lines indicate a promotion, dashed arrows represent unconfirmed events, and blunt arrows represent negative regulation. (**B**) Hormone signaling pathway genes known to be involved in the Pi deficiency-mediated regulation of PR growth in Arabidopsis and rice.

In summary, the molecular mechanisms by which low Pi inhibits PR growth are well studied in Arabidopsis (Figure 1). In rice, low Pi either promotes root growth or does not affect PR or CR growth, depending on the genetic background.

### 3.2. The Molecular Mechanisms Underlying the LR Response to Pi Limitation

Auxin acts as the major determinant in the Pi deficiency-induced establishment of the LR primordium and LR emergence [25]. The auxin receptor AtTIR1 and two auxin response factors, AtARF7 and AtARF19, are responsible for the increase in LR density under Pi-deficient conditions in Arabidopsis [53,71]. Under low-Pi conditions, the increased expression of *AtTIR1* triggers the degradation of the AUX/IAA proteins, releasing the ARF transcription factors to regulate the expression of the genes responsible for LR formation and emergence [53,72,73]. In addition, under low-Pi conditions, AtTIR1 upregulates *AtARF7* and *AtARF19* which, in turn, directly regulate the expression of *PHOSPHATE STARVATION RESPONSE 1* (*AtPHR1*), which encodes the core transcription factor regulating the expression of the P starvation response genes [74,75]. The impaired LR growth in the *arf7 arf19* double mutant under Pi-limited conditions could be partially rescued by constitutively expressing *AtPHR1*. Under Pi-limited conditions, the *phr1* mutants produced fewer LRs, while the *AtPHR1*-overexpressing plants displayed an increased LR number. These results suggest that the formation of LRs in response to Pi limitation is partially mediated by AtPHR1 in Arabidopsis.

Similarly, in rice, auxin plays an important role in LR remodeling under Pi starvation. The LRs of the *osarf16* mutant showed less of a response to Pi deficiency than those of the wild type, suggesting that OsARF16 positively regulates the LR response to Pi deficiency [76]. Under Pi-deficient conditions, the LR number and density underwent a greater increase in the *osarf12* mutants than in the wild type, suggesting that OsARF12 negatively regulates the LR response to Pi deficiency [77]. In addition, the phosphate transporter OsPHT1;8 affects Pi-dependent root growth, probably by regulating the distribution or polar transport of auxin [78]. This likely involved a feedback loop, as *OsPHT1;8* expression was in turn induced by IAA. Plants overexpressing *OsPHT1;8* produced more LRs than the wild type under Pi-replete conditions, but not under Pi-limited conditions. The *OsPIN* genes were upregulated in the *OsPHT1;8*-overexpressing plants under Pi-sufficient conditions, but not under Pi-limited conditions, further highlighting the interplay between auxin signaling and Pi signaling.

The SLs are also involved in regulating LR formation under low-Pi conditions in a MORE AXILLIARY BRANCHING 2 (AtMAX2)-dependent manner [23,79,80]. *AtMAX2* encodes an F-box protein, which is a part of the Skp-Cullin-F-box (SCF) E3 ligase complex. Mutants of *AtMAX2* (SL signaling) or *AtMAX4* (SL biosynthesis) showed a reduced LR response to low-Pi conditions in comparison with the wild type, suggesting that SLs play a role in Pi-dependent LR formation [79,80].

In rice, the SL biosynthesis genes *D10* and *D27* and the SL signaling gene *D3* (a homologous gene of *AtMAX2*) are responsible for regulating Pi-dependent LR formation [22]. Compared with the wild type, the *d10*, *d27*, or *d3* mutants showed a loss of sensitivity in the LR response to Pi deficiency. It was suggested that D3 regulates the transport of auxin from shoot to root by modifying the expression level of the *PIN* family genes [22]. Although SLs play an important role in Pi-dependent LR growth in rice, the detailed molecular mechanism underlying SL signaling in this process requires further investigation. In addition, a recent study found that Pi deficiency inhibits LR growth in mutants of *OsACS1*, which catalyzes the rate-limiting step in ethylene biosynthesis in rice, suggesting that ethylene is also involved in low-Pi-mediated LR growth [81].

**Figure 2:**
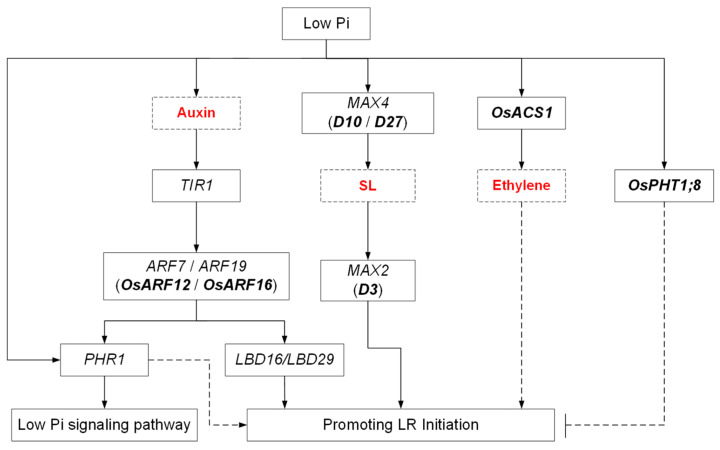
A working model of the molecular mechanisms underlying Pi starvation-induced LR growth in Arabidopsis and rice. Arrows with solid lines indicate a promotion, dashed arrows represent unconfirmed events, and blunt arrows represent negative regulation. Names in bold represent genes from rice. The hormones are highlighted in red.

Taken together, the above knowledge demonstrates that although the LR response to Pi limitation differs between rice and Arabidopsis, the underlying molecular mechanisms seem to be conserved between them (Figure 2).

### 3.3. The Molecular Mechanisms Underlying the RH Response to Pi Limitation

Auxin is essential for RH growth in Pi-limited Arabidopsis [25]. Knocking out *TRYPTOPHAN AMINOTRANSFERASE OF ARABIDOPSIS 1* (*AtTAA1*), an auxin biosynthesis gene, disrupted the promotion of RH by low-Pi conditions [25]. The RHs in the *ataux1* and *atpin2* mutants are not responsive to low-Pi conditions, suggesting that AtAUX1- and AtPIN2-mediated auxin distribution is essential for RH growth when Pi is limited [82]. Similarly, the RHs of *osaux1* were insensitive to the external Pi concentration, suggesting that the auxin transport pathway required for the RH response to Pi starvation is conserved in rice and Arabidopsis [83]. ROOT HAIR DEFECTIVE 6-LIKE 2 (AtRSL2) and AtRSL4 are basic helix-loop-helix (bHLH) family transcription factors, which play crucial roles in RH morphogenesis during the RH elongation stage [84,85]. This suggests that, under low-Pi conditions, auxin levels are upregulated at the root tip, increasing the *AtARF19*, *AtRSL2*, and *AtRSL4* expression levels to trigger RH elongation through the regulation of the various RH-related genes [25].

Recently, KARRIKIN INSENSITIVE 2 (AtKAI2) was reported to regulate the Pi starvation-promoted RH density and elongation through the ethylene signaling pathway [86]. AtKAI2 is an alpha/beta-hydrolase required for the plant response to karrikins, which are smoke-derived compounds mimicking endogenous signaling molecules [87]. AtKAI2 interacts with AtMAX2 to mediate the degradation of the target regulator SUPPRESSOR of MAX2 (AtSMAX2) in a proteasome-mediated manner [88]. Low-Pi availability increases the expression of *AtKAI2* and *AtMAX2* and then represses the expression of *AtACS7*, an ethylene precursor, subsequently triggering ethylene biosynthesis and signaling in the root via the degradation of AtSMAX1 and SMAX1-LIKE 2 (AtSMXL2) [86]. Subsequently, ethylene enhances AtAUX1 accumulation in the LR cap and epidermis, and AtPIN2 accumulation in the meristematic and elongation zones, resulting in RH elongation [86]. ETHYLENE INSENSITIVE 3 (AtEIN3), the master transcription factor in ethylene signaling, functions antagonistically with AtMYB30, a R2R3-MYB family transcription factor, in promoting RH growth under Pi deficiency [89,90]. The *atmyb30* mutant displayed longer RHs, while *AtMYB30*-overexpressing plants had shorter RHs than those of the wild type under Pi-limited conditions. By contrast, the RHs in *atein3-1* were shorter than those of the wild type under Pi deficiency. Furthermore, the RHs of *atmyb30-1 atein3-1* were shorter than those of *atmyb30-1* but longer than the *atein3-1* RHs under Pi-limited conditions, demonstrating the antagonistic relationship between AtMYB30 and AtEIN3. Another study indicated that AtMYB30 and AtEIN3 antagonistically regulate the transcription level of *AtRSL4* and other RH genes [90].

Cytokinins also play a vital role in Pi deficiency-induced RH growth [91,92]. The phosphoribohydrolase LONELY GUY 4 (AtLOG4) and its close homolog AtLOG3, which are rate-limiting enzymes in converting cytokinin into its bioactive form, function together to regulate Pi-dependent RH growth. *AtLOG3* and *AtLOG4* can be upregulated by the heterodimer transcription factor complex MONOPTEROS 5–LONESOME HIGHWAY (AtTMO5–LHW). Wendrich et al. further proposed that low Pi increases auxin signaling, which then induces localized cytokinin biosynthesis through the enhanced AtTMO5–LHW pathway. The biosynthesized cytokinin might then diffuse from the vasculature to the epidermal cells to modify their length and cell fate, contributing to RH growth [91].

SL regulates Pi starvation-promoted RH elongation through the same signaling pathway as LR growth in an AtMAX2-dependent manner [79,80]. In addition, the homeodomain protein ALFIN-LIKE 6 (AtAL6) is another key regulator of the RH response to Pi starvation [93]; the *atal6* mutant has shorter RHs than those of the wild type under Pi-limited conditions. Chandrika et al. suggested that AtAL6 might control RH elongation under Pi-limited conditions by regulating the expression of *ENHANCER OF TRY AND CPC1* (*AtETC1*), which functions in promoting the RH cell fate [93,94].

**Figure 3:**
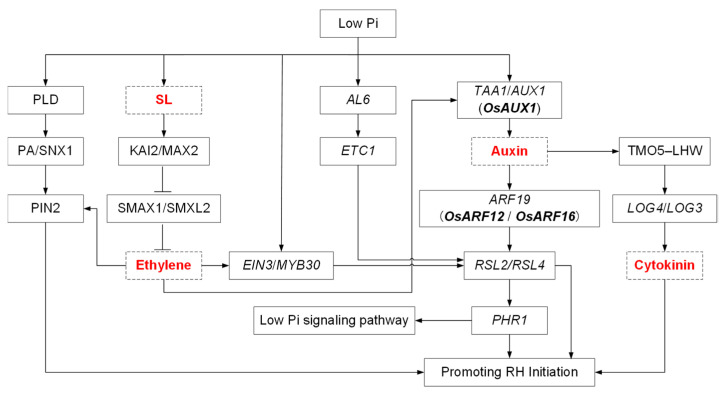
The molecular regulatory pathway of Pi starvation-induced RH growth in Arabidopsis and rice. Arrows indicate a positive regulation, and blunt arrows represent negative regulation. Names in bold represent genes from rice. The hormones are highlighted in red.

Taken together, Pi limitation affects RH growth through a completed regulatory network in Arabidopsis, which is summarized in Figure 3; however, the molecular mechanisms underlying Pi-dependent RH growth in rice have yet to be investigated.

### 3.4. Response of the RGA to Pi Limitation

A topsoil foraging root system containing shallower roots (greater RGA) is assumed to improve Pi acquisition efficiency in crops [95]. Pi deficiency promotes a greater RGA in an auxin-dependent manner in Arabidopsis [53,96]. This effect was not observed in the *attir1 atafb3* double mutant, however, indicating that the AtTIR1/AtAFB-mediated auxin signaling pathway plays an important role in RGA remodeling under Pi-deficient conditions [96]. Similarly in rice, Pi deficiency induces shallower root growth (bigger angles between the CRs and the direction of gravity). The rice actin-binding protein RICE MORPHOLOGY DETERMINANT 1 (OsRMD1) has been found to play a key role in regulating the CR growth angles in response to Pi [32]. OsRMD1 is localized on the statolith surface of the columella cells, controlling statolith sedimentation in response to gravitropic stimuli in the root tip by binding actin filaments and statoliths. The loss-of-function mutant *osrmd1* produced smaller RGAs caused by the faster movement of statoliths, making the mutant unresponsive to low Pi [32].

Greater RGAs contribute to improving the root surface area for Pi exploring; however, the molecular mechanisms underlying low-Pi-mediated RGA remodeling have yet to be investigated.

## 4. Hints for Improving Pi-Efficient Root Architecture in Crops

Improving crop Pi-use efficiency is urgently needed in modern agriculture due to the depletion of Pi ores, low P-use efficiency in crops, and increasing ecological concerns about excessive P use. Roots are the organ by which plants take up water and nutrients critical for growth and crop yields. The gramineous crops have a fibrous root system composed of PR, CRs, LRs, and RHs. Recently, Kuppe et al. revealed that, with hairs on the respective root types, the CRs were responsible for 48.8% of the total P uptake, while large LRs and small LRs (<1 cm length and <80 μm diameter) took up 20.6% and 30.6% of the total P uptake, respectively [97], suggesting that genotypes with more and longer CRs and LRs would be better for adapting to low-Pi soils. One example is the *OsPSTOL1*-overexpressing plants harbor larger root systems with more CRs and a higher root dry weight, which enables them to take up not only more Pi but also other nutrients, such as nitrogen and potassium, providing more nutrients for growth and yield [98,99]. Another example is DJ123, a variety with a much larger root system and high root vigor, which shows a better adaptation to low-Pi conditions and better responsiveness to fertilizer applications, with a relatively high grain yield in low-P African soils than Nerica4 (a popular rice variety in Africa) [100,101]. DJ123 contains twice the number of CRs, longer and denser RHs than the other nine tested modern rice varieties, which contributed to its superior Pi uptake under Pi-deficient conditions [100,102,103,104].

Pi largely accumulates in the topsoil in the field; therefore, plants with a large RGA are Pi uptake-efficient. The *quantitative trait locus for SOIL SURFACE ROOTING 1* (*OsqSOR1*) and *DEEP ROOTING 1* (*OsDRO1*) QTLs were found to be associated with the RGA via QTL mapping [105,106,107]. Oo et al. (2021) compared the growth of the near-isogenic lines (NILs) qsor1-NIL, Dro1-NIL, and IR64, which produced shallow, deep, and intermediate RGAs, respectively, in soils in which Pi accumulated in the surface layer. The qsor1-NIL plants with shallow root growth had the greatest biomass and Pi uptake, suggesting that a shallow root system is beneficial for rice Pi uptake [108].

Identifying the genes underlying the ideal root traits is important for breeding nutrient-efficient and high-yielding rice varieties; thus, it is important to identify the genotypic variations underlying changes in the numbers, lengths, and densities of the CRs, LRs, and RHs (known candidate genes are summarized in Table 1). Modern molecular breeding technologies can facilitate genome-wide elite allele selection, which in combination with gene editing and transgene techniques will greatly speed up the breeding of ideal root architectures to produce nutrient-efficient, high-yielding, and widely adaptable rice varieties. On the other hand, breeding a smart rice that can adjust its root system according to the soil nutrient levels could optimize the uptake and use efficiency of Pi or other nutrients.

## Figures and Tables

**Table 1:** List of known root-related genes affecting the adaptation to low Pi in rice.

Gene	Phenotype Compared with the Wild Type	Function Category	Reference
*OsWRKY74*	Longer PRs in *OsWRKY74*-overexpressing plants, shorter PRs in *OsWRKY74* RNAi plants under Pi deficiency	WRKY transcription factor	[49]
*OsAUX4*	*osaux4* produced a shorter PR under Pi-replete conditions but was insensitive to Pi deficiency	Auxin influx carrier	[50]
*OsAUX1*	*osaux1* produced shorter RHs under Pi-replete conditions but was insensitive to Pi deficiency	Auxin influx carrier	[83]
*OsPIN1b*	*ospin1b* produced shorter PRs and CRs under Pi-replete conditions but was insensitive to Pi deficiency	Auxin efflux transporter	[51]
*OsARF12*	*osarf12* produced more CRs, an increased number and density of LRs, and longer RHs under Pi-limited conditions	Auxin response factor	[52]
*OsARF16*	*osarf16* produced fewer LRs and shorter RHs under Pi-limited conditions	Auxin response factor	[76]
*OsMYB1*	*osmyb1* produced a longer PR under Pi-limited conditions	MYB transcription factor	[67]
*LTN1*	*ltn1* produced longer PRs and CRs under Pi-limited conditions	A ubiquitin-conjugase	[70]
*OsPHT1;8*	*OsPHT1;8*-overexpressing plants produced shorter PRs and CRs, but more LRs and RHs under Pi-replete conditions, but were insensitive to Pi deficiency	Phosphate transporter	[78]
*D10/D27*	*d10*, *d27* mutants produced shorter PRs and CRs and more LRs under Pi-replete conditions but were less sensitive to Pi deficiency	SL biosynthesis genes	[22]
*D3*	*d3* produced shorter PRs and CRs and more LRs under Pi-sufficiency but was less sensitive to Pi deficiency	SL signaling gene	[22]
*OsACS1*	*osacs1* produced fewer LRs under Pi-replete conditions but was less sensitive to Pi deficiency	Ethylene biosynthesis gene	[81]
*OsRMD1*	*osrmd1* had a smaller RGA	An actin-binding protein	[32]
*OsPSTOL1*	*PSTOL1*-overexpressing plants produced a larger root system with more CRs and a higher root dry weight, as well as an enhanced grain yield in the IR64 and Nipponbare backgrounds in Pi-deficient soils	Protein kinase	[98,99]
*OsqSOR1*	Plants containing the elite allele at this QTL produced shallow roots with a greater biomass and Pi uptake than the other two lines, Dro1-NIL and IR64, which had deep and intermediate RGAs, respectively	A homolog of DRO1 that functions downstream of the auxin signaling pathway	[108]

## Data Availability

All relevant data can be found within the manuscript.
